# Data for the evaluation of groundwater quality using water quality index and regression analysis in parts of Nalgonda district, Telangana, Southern India

**DOI:** 10.1016/j.dib.2020.106235

**Published:** 2020-08-27

**Authors:** K. Saikrishna, D. Purushotham, V. Sunitha, Y. Sudharshan Reddy, D. Linga, B. Kiran Kumar

**Affiliations:** a Department of Geology, Kakatiya University, Warangal 506009, Telangana, India; b CSIR-National Geophysical Research Institute, Hyderabad 500007, Telangana, India; c Department of Geology, Yogi Vemana University, Kadapa 516005, India

**Keywords:** Groundwater, WQI, Correlation coefficients, Regression analysis, Linear plots

## Abstract

The main focus of this study is to evaluate the groundwater quality through drinking water quality index and regression analysis in semi-arid region and the results are examined with reference to the drinking water quality standards laid down by WHO. Water quality index (WQI) was determined from fourteen physicochemical parameters like pH, EC, TDS, total hardness, total alkalinity, sodium, potassium, calcium, magnesium, chloride, bromide, nitrate, sulphate and fluoride. The drinking water quality index values range from 32.8 - 442.4, indicating three categories i.e. poor, very poor and unfit, which are inappropriate for drinking. Regarding correlation analysis results, EC, TDS, TH, Na^+^, Mg^2+^, Ca^2+^ and Cl^−^ shows high correlation. Most of the parameters are more or less correlated with each other, regression relations have the same correlation coefficients and pH, Na^+^, EC, TDS, Mg^2+^, Ca^2+^, Cl^−^, SO_4_^2−^, CO_3_^2−^, TH were significantly positively correlated (R>0.9), indicate the increase in the pollution load.

## Specifications Table

**Table:** 

Subject area	Hydro Chemistry
More specific subject area	Water Quality
Type of data	Tables and figures
How data was acquired	GPS was used to mark the location and Ion chromatography was employed to analyses for various parameters such as pH, electrical conductivity (EC), Total dissolved solids (TDS), TH, Calcium (Ca^2+^), Magnesium (Mg^2+^), Sodium (Na^+^), Potassium (K^+^), Carbonate (CO_3_^2-)^, Bicarbonate (HCO_3_^−)^, Chloride (Cl^−^), Sulfate (SO_4_^2−^) and Nitrate (NO_3_^−)^ ions. Ion Chromatography is employed to analyze the cations and anions. The columns used were AS-14 and CS-17 for anions and cations, respectively. Carbonates and bicarbonates were measured by end-point titration method. ARC GIS 10.3 for producing distribution maps [1].
Data format	Raw and analyzed
Parameters for data collection	Samples of ground water were collected in two litre bottles and stored in dark room under specified conditions. To analyse the concentration levels of various physico chemical Parameters using standard methods.
Description of the data collection	Forty Ground Water Samples were collected in different bore wells from different parts of Nalgonda District, Telanagana analysed for pH, Electrical Conductivity, Total dissolved solids, Total Hardness, Total alkalinity, Calcium, Magnesium, Potassium, Sodium, Chloride, Bromide, Sulphate, and Fluoride followed by APHA guidelines and W.H.O standard limits [2], [3], [4].
Data source Location	Nalgonda District, Telanagana State
Data accessibility	Data is included in this Article

## Value of the Data

• The data represented is used to calculate water quality index which aids in assessment of groundwater in around semi-arid region, can help to better understanding the quality of groundwater and taking necessary steps to regular monitoring to avoid groundwater contamination and suggest proper remediation technologies.
• The linear regression analyses are used for the water quality parameters and it measure higher and better levels of significance in their correlation coefficient. The systematic calculation of regression analysis provides indirect means for the fast monitoring of water quality.
• Due to limited studies in the study region, this data would be useful for the researches, government and nongovernmental organizations to adopt effective planning methods and mitigation measures in limestone mining areas and aids in sustainable development of groundwater.

## 1 Data Description

### 1.1 Study area

**Fig. 1:**
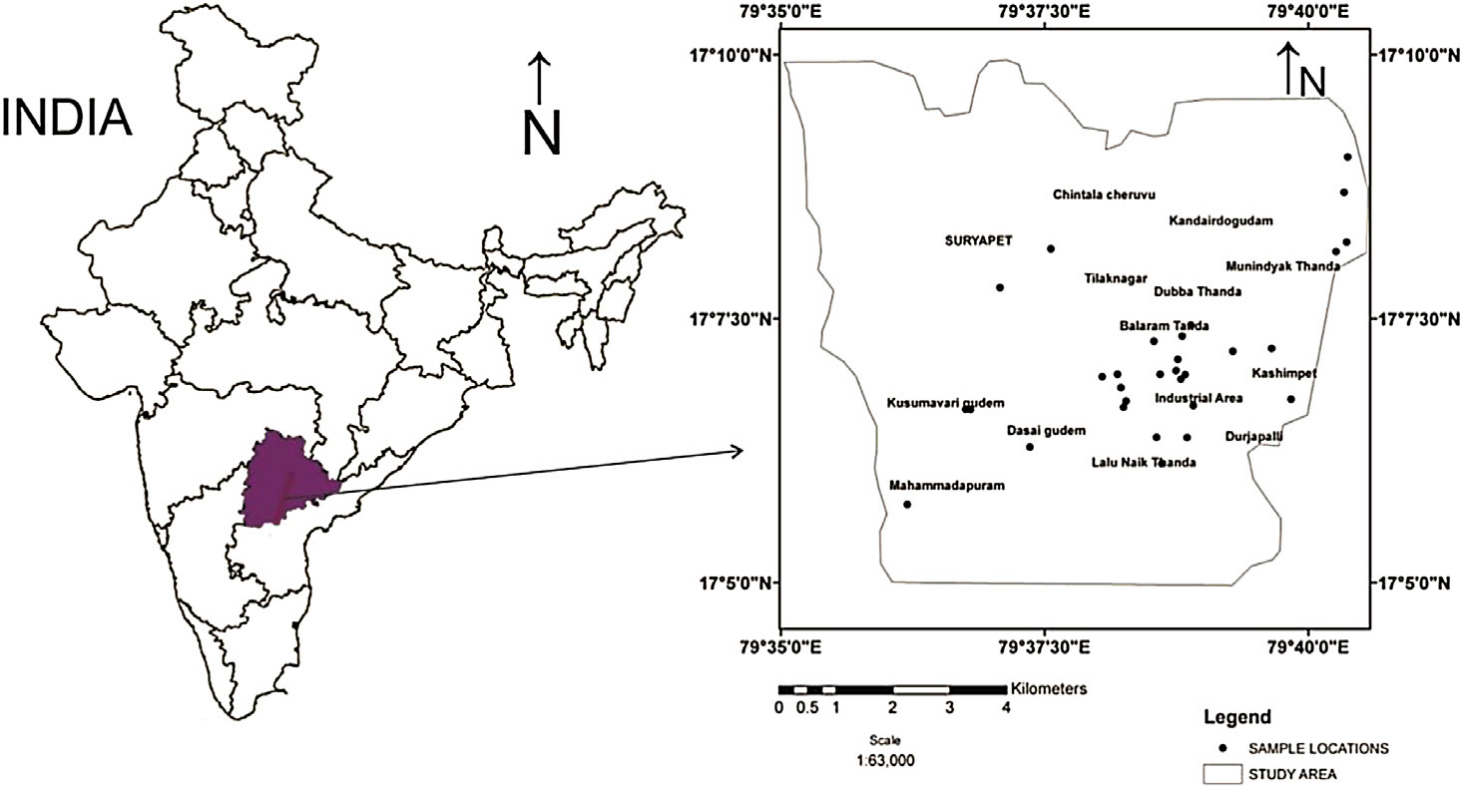
Location map of the study area.

**Fig. 2:**
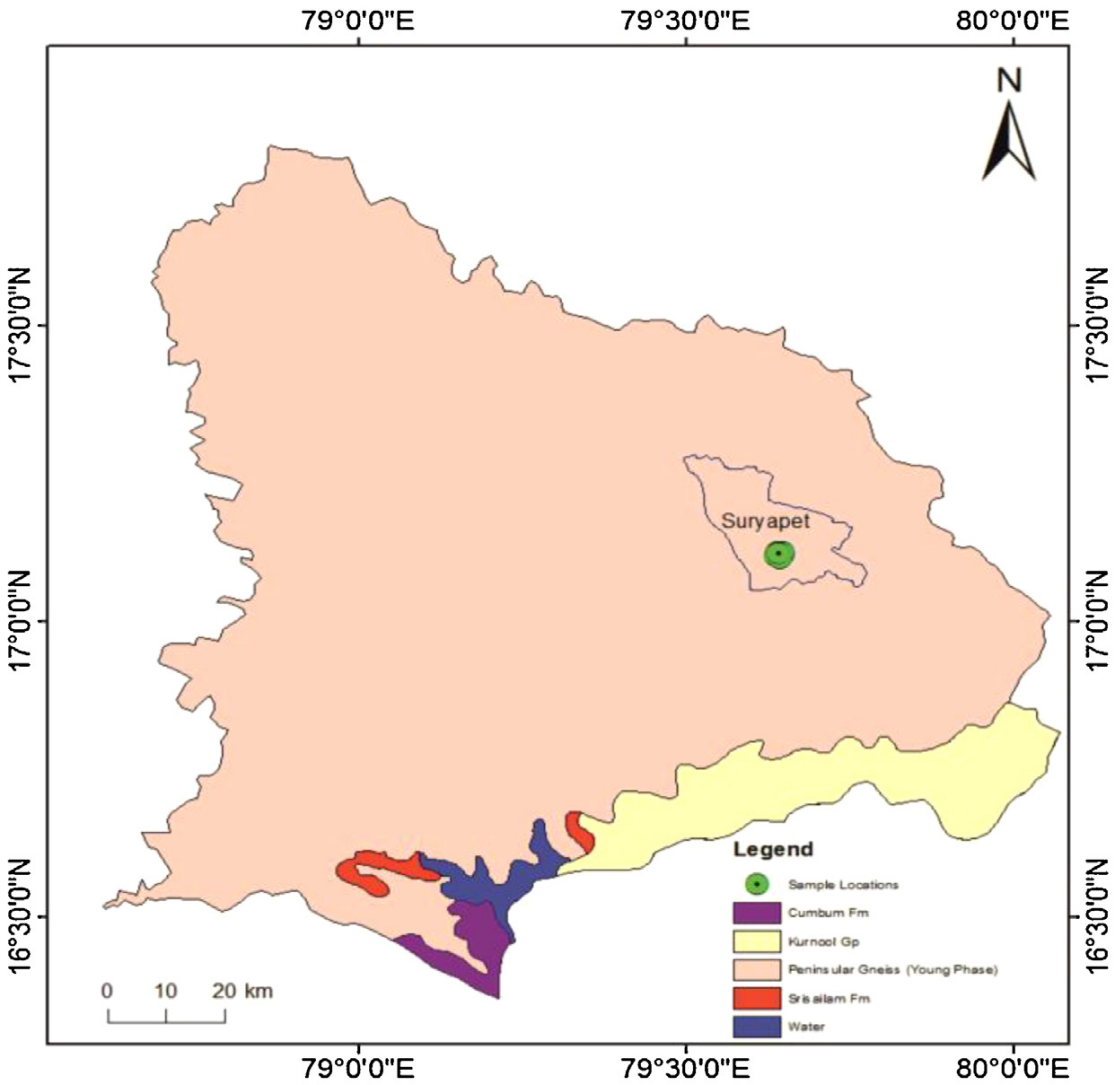
Geology map of the study area.

The area situated between 79°35’30” E and 17°05’00” N to 79°40’35” E and 17°10’00” N latitude (Fig. 1) and located in and around Suryapet city of Nalgonda district, Telangana state. The study area experiences the semi-arid climate; where pollution and scarcity of groundwater resources has been observed due to anthropogenic activities and it is very significant in terms of rapid industrialization, pesticide usage, pharmaceutical, agro based industries, urban development and granite polishing [[5], [6], [7], [8]]. Geologically, the study area is essentially composed of Archaean crystalline rocks such as granites/granite gneisses, and the dolerite dykes, pegmatite veins and quartz veins are intruded into the Archaean basement rock. The Cuddapah and Kurnool system is represented by consolidated meta sedimentary rocks like limestones, quartzites and shales in the southern part of the Nalgonda district (Fig. 2). The groundwater occurs in the crystalline aquifer at depths ranging from 6–15 m and in dug-cum-bore wells at a depth of 60m. The yield of irrigation wells range from 100 to150 m^3^/day. The fractures are randomly oriented and are observed at depth of 40–60 m.

### 1.2 Data

**Fig. 3:**
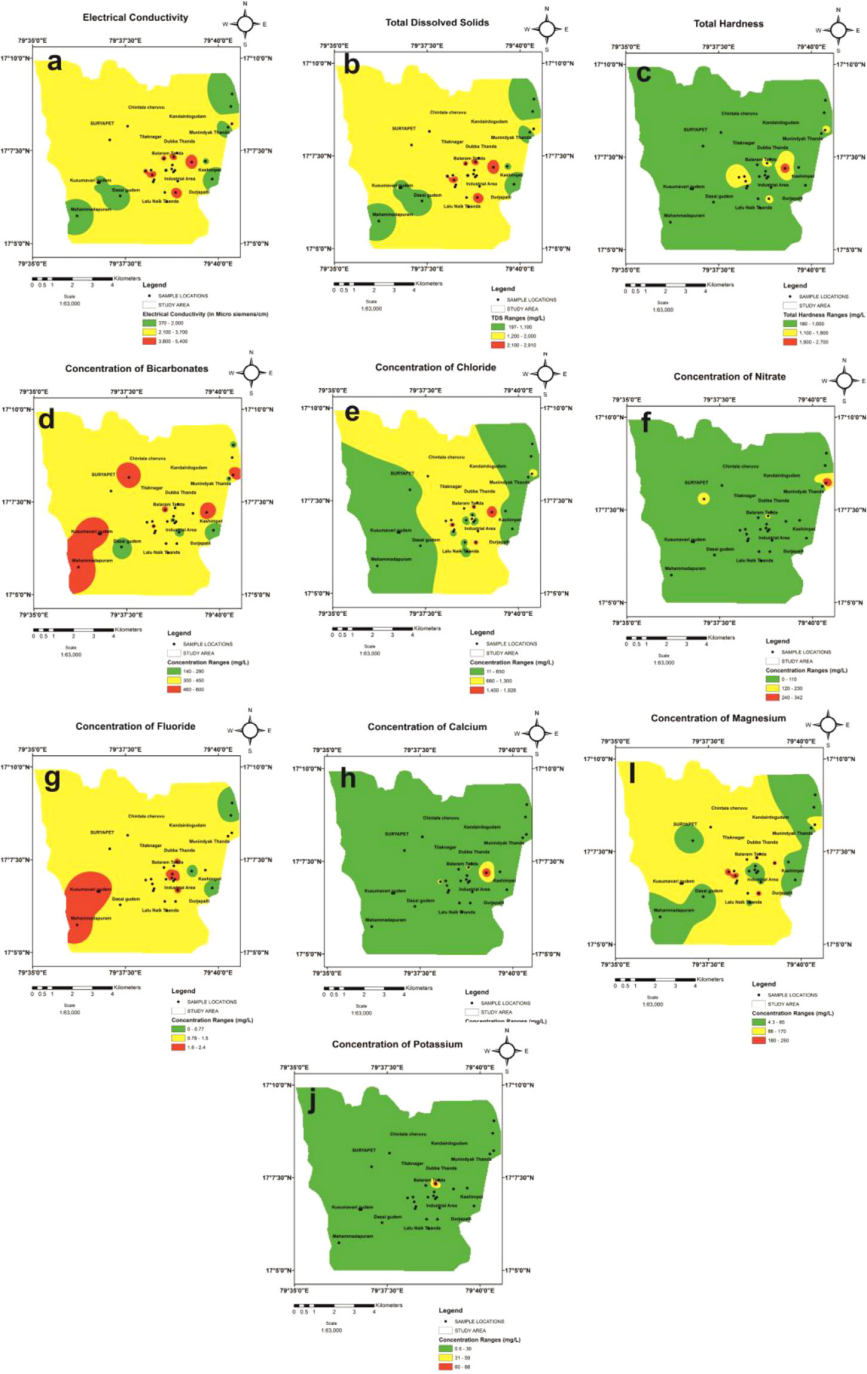
Spatial variation maps of physico-chemical parameters (3a to 3j).

**Fig. 4:**
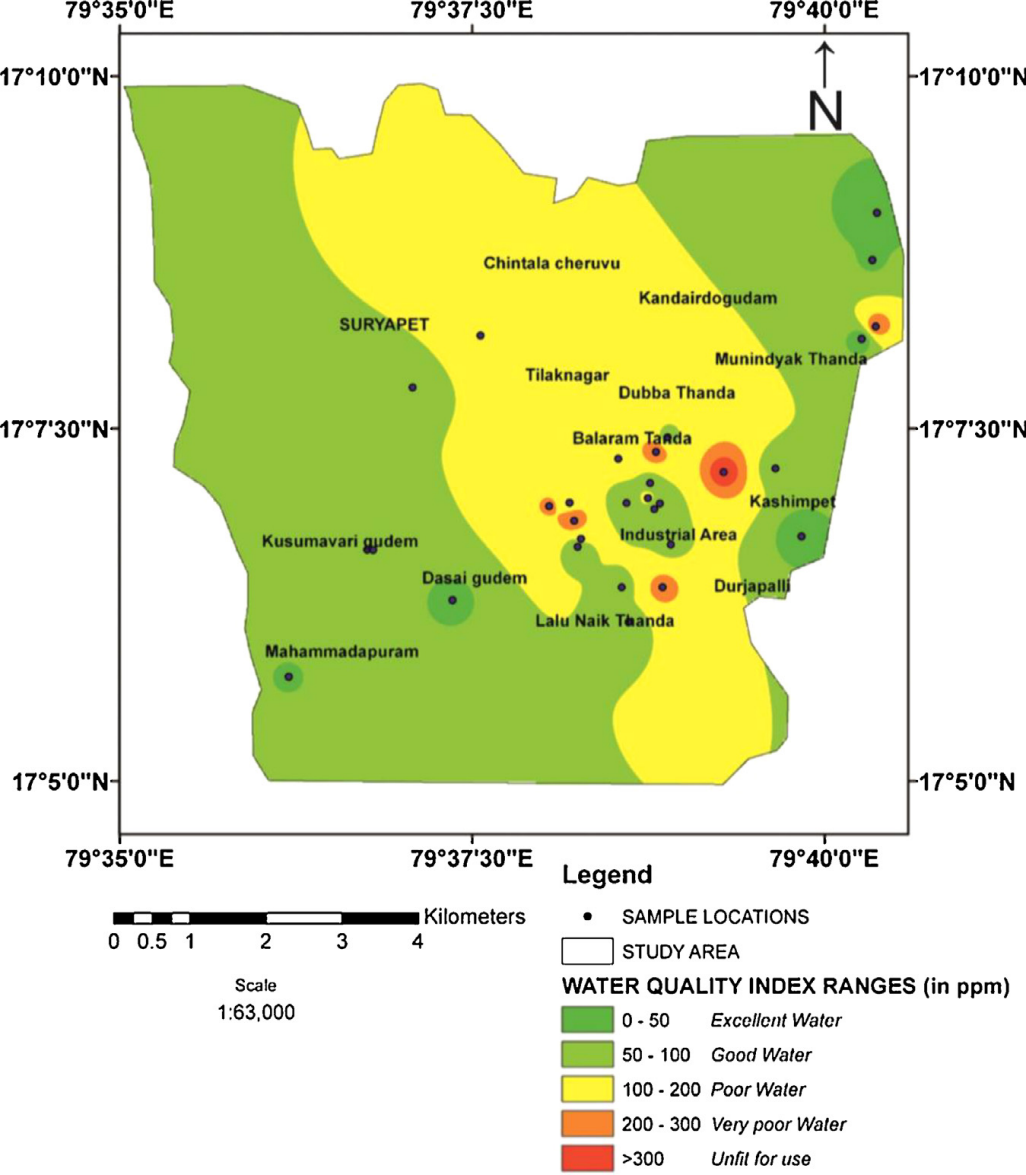
Water quality classification based on WQI values of the study area.

**Fig. 5:**
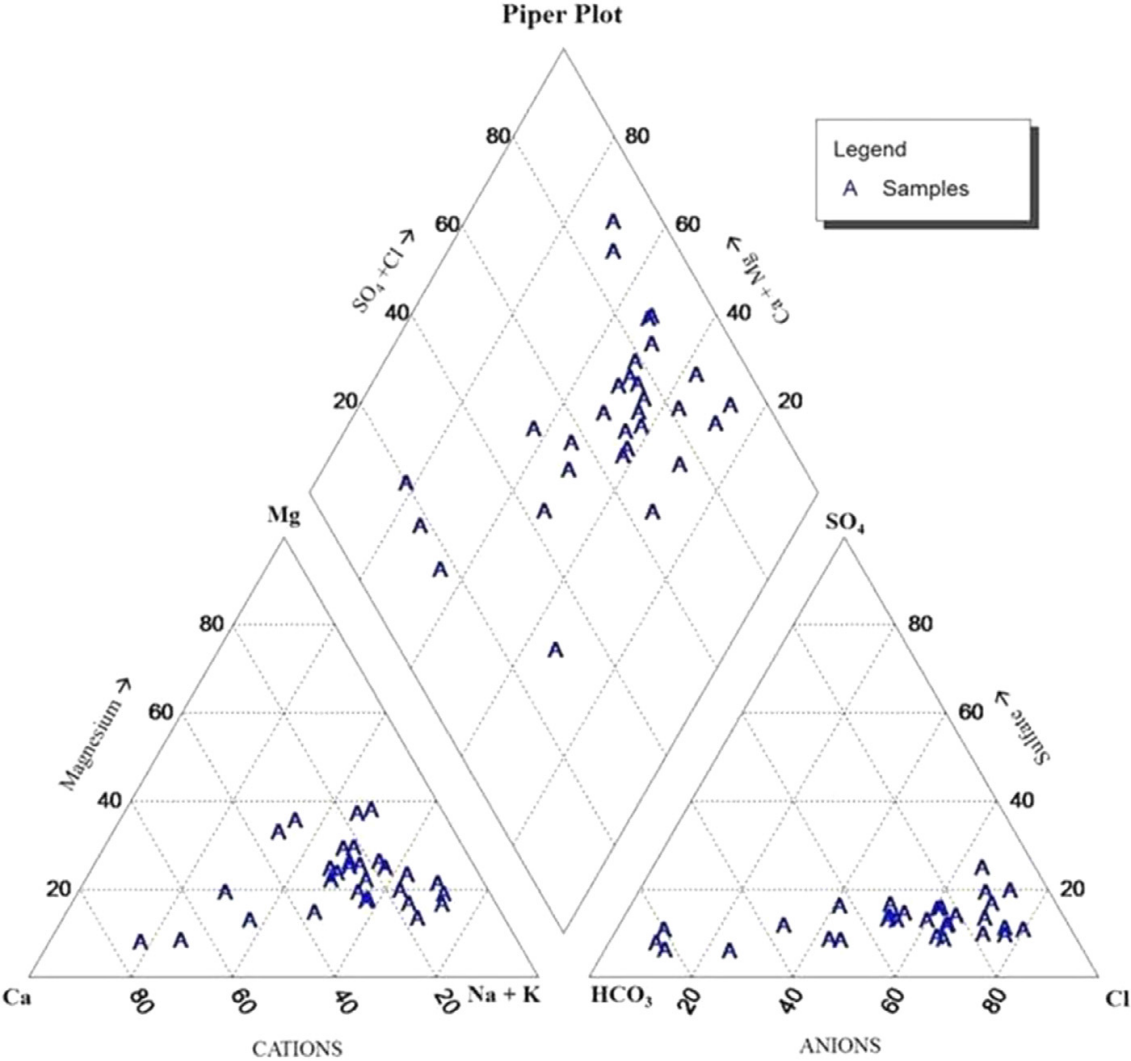
Piper tri linear diagram representing the chemical analysis of the study area.

**Fig. 6:**
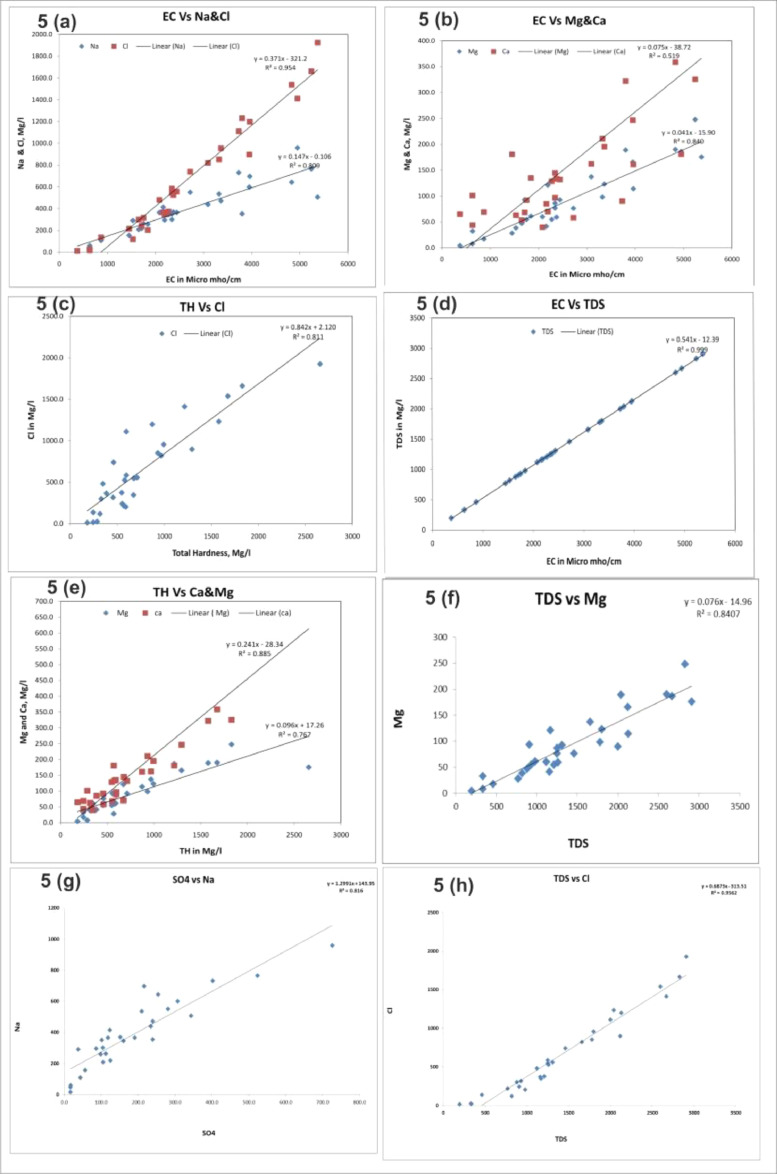
Linear plot between EC Vs Na & Cl; EC Vs Mg & Ca; TH Vs Cl; EC Vs TDS; TH Vs Ca & Mg; TDS Vs Mg; SO4 Vs Na; TDS Vs Cl.

Normal statistics of water quality parameters of groundwater samples were indicated in Table 1. WHO standards weight (W i) and calculated relative weight (Wi) for each parameters are shown in Table 2. Spatial variation maps of physico-chemical parameters (3a to 3n) are shown in Fig. 3. Water quality classification based on WQI values of the study area were depicted in Table 3 and Fig. 4. Fig. 5 represents Piper tri linear diagram representing the chemical analysis of suryapet region. Correlation Matrices for water quality parameters are shown in Table 4. Higher and lower levels of significant correlation coefficient values of the parameters are shown in Table 5 and Linear plots of various parameters are shown in Fig. 6.

**Table 1:** Normal statistics of water quality parameters of groundwater samples.

Water Quality parameters	pH	EC	TDS	TH	Ca	Mg	Na	K	NO_3_	Cl	F	SO_4_	HCO_3_	CO_3_
Minimum	7.1	371	197	180	39.8	4.3	14.5	0.9	1.1	11.6	0.2	16.8	140	0
Maximum	8.2	5370	2910	2656	774.5	246.4	958	87.5	342	1925	2.3	726	600	80
Mean	7.8	2631	1411	777	159.6	92.3	389	8.42	50	657	1	188	383.1	27
Standard Deviation	0.30	1377	745	560	144.02	61.7	226	17	72	524	0.5	157	124.1	31

**Table 2:** WHO standards weight (Wi) and calculated relative weight (W_i_) for each parameters.

Parameters	Standard Permissible Value (S)i WHO, 2004)	Weight (Wi)	Relative Weight (Wi)	Quality Rating Scale
pH	6.5-8.5	4	0.074	242
TDS	500	4	0.068	80
EC	500	4	0.086	149.4
TH	300	5	0.086	80.4
Calcium	75	4	0.086	290.5
Magnesium	50	4	0.068	103.2
Sodium	200	4	0.068	171.4
Potassium	200	2	0.068	182.7
Nitrate	45	5	0.068	172.3
Chloride	250	5	0.034	2.5
Sulphate	250	5	0.068	102.9
Bicarbonate	500	4	0.086	182
Carbonate	500	4	0.068	454
Fluoride	1-1.5	4	0.068	0

**Table 3:** Water quality classification based on WQI values of the study area.

Water Quality	WQI Values	WQI of samples	No. of samples	Percentage (%)
Excellent water	<50	32.8	1	3
Good water	50-100	57.3 - 77.4	3	10
Poor water	100-200	106.3 - 174.4	15	50
Very poor water	200-300	216.5 - 293	6	20
unfit for use	>300	336.6 - 442.3	5	17

**Table 4:** Correlation matrices for water quality parameters.

Parameters	pH	Cond	TDS	TH	Na	K	Mg	Ca	Cl	SO4	HCO3	CO3	NO3	F
pH	1													
Cond	-0.14091	1												
TDS	-0.14158	0.999971	1											
TH	-0.24602	0.877071	0.878622	1										
Na	-0.02559	0.899596	0.898788	0.592835	1									
K	0.187207	0.37926	-0.98652	0.26959	0.366135	1								
Mg	-0.1729	0.916914	0.916922	0.481412	0.760275	0.315835	1							
Ca	-0.26133	0.720677	0.723086	0.940815	0.388339	0.198037	-1.71359	1						
Cl	-0.18044	0.977045	0.977855	0.900906	0.836251	0.396248	0.890349	0.776451	1					
SO4	-0.13214	0.855549	0.855892	0.645045	0.903321	0.231364	0.785495	0.451925	0.820503	1				
HCO3	0.048215	0.331011	0.328901	0.31774	0.281743	-0.05759	0.400371	0.213003	0.178476	0.099973	1			
CO3	0.756907	-0.16918	0.16962	-0.24715	-0.06131	0.189177	-0.10402	-0.31146	-0.23942	-0.12707	0.101139	1		
NO3	-0.03062	0.224467	0.223416	0.29451	0.135169	0.305117	0.21006	0.311069	0.13663	0.054493	0.391088	0.023009	1	
F	0.335663	0.216923	0.216622	0.022457	0.332867	0.016788	0.177523	-0.09003	0.12321	0.154757	0.328628	0.470655	- 0.03301	1

**Table 5:** Least square of the relation (Y = AX + B) among significantly correlate parameters.

Y	X	r	a	b	Regression Equation	R square
CO_3_^−^	pH	**0.75**	-566.5	75.96	CO_3_^−^ = 75.96(PH) - 566.5	0.57
TDS	EC	**1.00**	-12.39	0.54	TDS = 0.541(EC) - 12.39	0.99
TH	EC	**0.87**	-161.9	0.35	TH= 0.357(EC) - 161.9	0.76
TH	TDS	**0.87**	-155.3	0.66	TH= 0.660(TDS) - 155.3	0.77
Na^2+^	EC	**0.90**	-0.106	0.14	Na^2+^ = 0.147(EC) - 0.106	0.80
Na^2+^	TDS	**0.89**	3.635	0.27	Na^2+^= 0.272(TDS) + 3.635	0.80
Mg^2+^	EC	**0.91**	-15.9	0.04	Mg^2+^ = 0.041(EC) - 15.90	0.84
Mg^2+^	TDS	**0.91**	-14.96	0.07	Mg^2+^= 0.076(TDS) - 14.96	0.84
Mg^2+^	Na^2+^	**0.76**	11.61	0.20	Mg^2+^ = 0.207(Na)+ 11.61	0.57
Ca^2+^	EC	**0.72**	-38.72	0.07	Ca^2+^= 0.075(EC)- 38.72	0.51
Ca^2+^	TH	**0.94**	-28.34	0.24	Ca^2+^= 0.241(TH) - 28.34	0.88
Cl^−^	EC	**0.97**	-321.2	0.37	Cl^−^= 0.371(EC)- 321.2	0.95
Cl^−^	TDS	**0.97**	-313.5	0.68	Cl^−^ = 0.687(TDS) - 313.5	0.95
Cl^−^	TH	**0.90**	2.12	0.84	Cl^−^ = 0.842(TH) + 2.120	0.81
Cl^−^		**0.83**	-96.08	1.93	Cl^−^= 1.936(Na) - 96.08	0.69
Cl^−^	Mg^2+^	**0.89**	-40.33	7.55	Cl^−^= 7.551(Mg) - 40.33	0.79
Cl^−^	Ca^2+^	**0.77**	205.9	2.82	Cl^−^= 2.823(Ca)+ 205.9	0.60
SO_4_^−^	EC	**0.85**	-68.71	0.09	SO_4_^−^ = 0.097(EC) - 68.71	0.73
SO_4_^−^	TDS	**0.85**	-66.56	0.18	SO_4_^−^= 0.180(TDS)- 66.56	0.73
SO_4_^−^	Na^2+^	**0.90**	-55.74	0.62	SO_4_^−^ = 0.628(Na) - 55.74	0.81
SO_4_^−^	Mg^2+^	**0.78**	3.781	2	SO_4_^−^ = 2.000(Mg)+ 3.781	0.61
SO_4_^−^	Cl	**0.82**	26.64	0.24	SO_4_^−^= 0.246(Cl)+ 26.64	0.67

## 2 Experimental design, methods and materials

### 2.1 Materials and methods

Groundwater samples were collected from thirty locations (∼10 to 40 m deep) in post monsoon season. GPS was used to mark the location and ion chromatography was employed to analyze for various ionic and non-ionic parameters such as pH, EC (electrical conductivity), TDS (Total dissolved solids), TH (Total hardness), Cations and Anions (Fig. 3a to 3j). Total fourteen parameters were considered to generate the Drinking Water Quality Index (DWQI), correlation analysis, correlation regression study along with water quality characteristics were determined.

### 2.2 Analytical procedures

WQI was calculated using the World Health Organization standards [2] and Indian Standards [3] in the following steps. Water quality index method for groundwater quality assessment is widely used around the world for assessment & management of groundwater [4–7]. The WQI calculation was carried out using a weighted arithmetic index as shown below [9, 10].

For computing WQI four steps are followed:

The groundwater experimental study (WQI) (Fig. 5) by means of fourteen physical and chemical parameters of the study area recognize with the purpose of water quality for drinking purpose, values range from 32.8 - 442.4, (Table 3) indicating three categories viz. poor, very poor and unfit.

**Step: 1**

The 14 parameters are allocated a weight (wi) and calculated relative weight (Wi) for each parameter of drinking water purposes is given in Table 1. Nitrate assigned by weight (Max) of five parameters because their major significance in water quality assessment [11]. As per significance in the complete quality of water for drinking purposes, the Na^+^, K^+^, Ca^2+^ and Mg^2+^ were allocated a weight between one and five.

**Step: 2**

The following equation is used to calculate the virtual weight (Wi), which is derived from weighed arithmetic index formula [10, 12–13]

$$\begin{array}{c} Wi=wi/\sum^{n}\;wi\\ \;\;\;\;\;\;\;\;\;\;\;\;\;i=1 \end{array}$$

Relative weight =Wi

Weight of each parameter = wi

Number of parameters = n

**Step: 3**

Qi=(Ci/Si)×100

Calculated each parameter concentration of each water sample = (Qi)

Relevant standard = (Ci) and then multiplied by 100 = Si

**Step: 4**

Sub Index (SIi) is determined for each chemical parameter

SIi=Wi×Qi

**Correlation Coefficient (r):**

The correlation coefficient **r** equation

$$r=\frac{n\Sigma xy\;\Sigma -x\;\Sigma y}{\surd \left[n\Sigma x2--\left(\Sigma x\right)2\right]\;\;\left[n\Sigma y2--\left(\Sigma y\right)2\right]}$$

**x** and **y** be any two variables, (Xi, Yi) be **n** pairs of identified values of (I =1, 2, 3……**n**) variables and in equation **r** between the variables x and y.

Where, the summations are taken above 1 to n (n=number of observations). The values of observed parameters ‘a’ and ‘b’ were considered with the help of Eqs. (2) and (3).

$$a=\frac{n\Sigma xy-\Sigma x\;\Sigma y}{n\Sigma x^{2}--{\left(\Sigma x\right)}^{2}}$$

The regression analysis has been performed using by SPSS 11.0 Statistical Software.

**Regression equation**

Y=ax+b

SPSS 18 software used to study the correlation among different water quality parameters and the regression analysis.

It assesses the degree of association that exists among two variables, which shows how one variable predicts the other; it can be used for swift monitoring of water quality. A high correlation coefficient (nearly 1 or -1) reveals that the relationship between two variables is good. If zero is recorded it means that there is no relationship between two variables, positive values show a positive relationship while negative values of ‘r’ indicate an inverse relationship (Table 4).

The statistical analysis results are given in the Table 3, which indicate (i) pH has a positive correlation with CO_3_^2−^_,_ F^−^, K^+^; weak correlation with HCO_3_^2-;^ negative correlation with EC, TDS, TH, Na^+^, Mg^2+^, Ca^2+^, Cl^−^, SO_4_^2−^_,_ NO_3_^−^_,_ HCO_3_^−^ and F^−^ (ii) EC shows positive correlation with TDS, TH, Na^+^, K^+^, Mg^2+^, Ca^2+^, Cl^−^, SO_4_^2−^_;_ weak correlation with HCO_3_^−^, NO_3_^−^, K^+^ and F^−^; negative correlation with CO_3_^2−^ (iii) TDS shows positive correlation with TH, Na^+^, K^+^, Mg^2+^, Ca^2+^, Cl^−^, SO_4_^2−^; weak correlation with HCO_3_^−^, NO_3_^−^, F^−^, CO_3_^2−^; negative correlation with K (iv) TH has positive correlation with Na^+^, K^+^, Mg^2+^, Ca^2+^, Cl^−^, SO_4_^2−^_;_ weak correlation with NO_3_^−^, F^−^, HCO_3_^−^; negative correlation with CO_3_^2-^ (vi) Mg^2+^ has positive correlation with Cl^−^, SO_4_^2−^, HCO_3_^−^; weak correlation with NO_3_^−^, F^−^; negative correlation with Ca^2+^, CO_3_^2−^_._ (vii) Ca has positive correlation with Cl^−^, SO_4_^2−^_;_ weak correlation with HCO_3_^−^, NO_3_^−^; negative correlation with F^−^, CO_3_^2−^ (viii) Cl^−^ has positive correlation with SO_4_^−^; weak correlation with HCO_3_^−^, NO_3_^−^, F^−^; negative correlation with CO_3_^2−^ (ix) SO_4_^2−^weak correlation with HCO_3_^−^, NO_3_^−^, F^−^; negative correlation with CO_3_^2−^ (x) HCO_3_^−^, weak correlation with NO_3_^−^, F^−^. CO_3_^2−^ weak correlation with NO_3_^−^, F^−^. NO_3_^−^ negative correlation with F^−^.

## Linear regression analyses

The linear regression analyses are used for the water quality parameters that square measure found to possess higher and better levels of significance in their correlation coefficient. The systematic calculation of regression analysis provides indirect means for the fast monitoring of water quality. In the correlation regression study, majority of the parameters are almost correlated with each other respectively. The correlation between TDS-EC; TH-EC; TH-TDS; Na^2+−^EC; Na^2+^-TDS; Mg^2+^-EC; Mg^2+^-TDS; Ca^2+−^TH; Ca^2+−^EC; Cl^−^-TDS; Cl^−^-TH; Cl^—^Na^+^; Cl^—^Mg^2+^; SO_4_^2−^-EC; SO_4_^2−^–TDS; SO_4_^2−^ -Na; SO_4_^2−^- Cl^−^ is positive. It is evident that the pH, Na^2+^, EC, TDS, Mg^2+^, Ca^2+^, Cl^−^, SO_4_^2−^, CO_3_^2−^ TH were significantly and positively correlated (R>0.9).

## Declaration of Competing Interest

None.

## Appendix Supplementary materials

Image, application 1

Image, application 2
